# HMGB1 Mediated Inflammation and Autophagy Contribute to Endometriosis

**DOI:** 10.3389/fendo.2021.616696

**Published:** 2021-03-19

**Authors:** Jingying Huang, Xuan Chen, Yuchun Lv

**Affiliations:** Department of Obstetrics and Gynecology, Quanzhou First Hospital Affiliated to Fujian Medical University, Quanzhou, China

**Keywords:** HMGB1, inflammatory response, autophagy, endometriosis, hypoxia

## Abstract

**Aim:**

High mobility group box (HMGB)-1 has been implicated in endometriosis due to the important regulatory roles of inflammation in endometriosis. The aim of the present study was to explore the roles of HMGB-1 in endometriosis and to elucidate the underlying mechanism.

**Methods:**

Endometrial specimens were collected from women with endometriosis and healthy volunteers. Immunohistochemistry staining was used to determine the expression patterns and localization of HMGB-1 in the normal, eutopic and ectopic endometrial tissues. Western blotting and qRT-PCR were used to determine the mRNA and protein levels of inflammatory cytokines [interleukin (IL)-6, tumor necrosis factor (TNF)-α and IL-1β], autophagy-related markers [beclin-1, autophagy-related (atg)13, microtubule-associated protein light chain (LC)3-I, LC-II and p62] and HMGB-1, respectively. Spearman’s rank correlation analysis was employed to investigate the correlation between HMGB-1 with inflammatory cytokines and beclin-1. Besides, human endometrial stromal cells (HESCs) were isolated from ectopic endometrium and subsequently transfected with shRNA against HMGB-1. After the transfected cells were subjected to hypoxia, ELISA was used to determine the levels of HMGB-1 and inflammatory cytokines in the cell supernatant. Western blotting was used to determine the expression levels of autophagy-related markers in the cells.

**Results:**

Positive correlations were observed between HMGB-1 and the inflammatory cytokines. In addition, a positive correlation was also identified between HMGB-1 and beclin-1 in the ectopic endometrium. Further results demonstrated that autophagy-related markers beclin-1, atg13 and p62 were significantly upregulated in the ectopic endometrium. In addition, HMGB-1 knockdown suppressed the levels of inflammatory cytokines IL-6, TNF-α and IL-1β and autophagy-related markers beclin-1 and atg13, while upregulated p62 in HESCs under hypoxic condition.

**Conclusion:**

Knockdown of HMGB-1 under hypoxic condition regulated inflammatory cytokines and autophagy-related markers. HMGB-1 might contribute to the development of endometriosis in part through regulating inflammatory response and autophagy.

## Introduction

Endometriosis is a chronic disorder of the endometrium (1, 2). The abnormal growth and infiltration of endometrial cells, including endometrial epithelial cells and stromal cells in the endometrium, into the deep endometriosis tissues causes the formation of nodules and masses in the endometrium (2, 3). Endometriosis is one of the most important risk factors of chronic pelvic pain, dysmenorrhea and infertility (4, 5). Endometriosis affects around 15% of women of reproductive age, while 30 to 50% of infertile women suffer from endometriosis (6). Inflammation plays an important role in endometriosis (4). Elevated inflammatory cytokines and mediators in the peritoneal fluid, which are frequently found in patients with endometriosis (7), induce endometriotic symptoms.

Autophagy is known as a housekeeping regulator for maintaining cellular homeostasis by removing unnecessary or dysfunctional components (8). Autophagy is activated when cells undergo stresses such as endoplasmic reticulum stress and nutrient deprivation (9). Autophagy have been implicated to play important roles in a series of diseases including aging, Parkinson’s disease, endometriosis and cancers (10, 11). One study demonstrated that autophagy was suppressed in the eutopic and ectopic endometrium from women with endometriosis (12). This study also revealed that autophagy was closely linked with endometriosis (12). The roles of autophagy are controversial in the endometriosis. Yang and colleagues reported that autophagy was shown to be suppressed in human endometrial stromal cells (HESCs) of endometriotic tissue (13). However, some other studies reported that autophagy is upregulated in endometriosis and hypoxia induces autophagy (14, 15). In fact, many studies have supported that autophagy affects endometriosis in part by regulating the proliferation and apoptosis of HESCs (16, 17). It is important to discover novel strategies against endometriosis by the regulation of autophagy.

HMGB-1 is a non-histone DNA binding protein and is secreted by many types of immune cells, including monocytes, macrophages, and dendritic cells (18). HMGB-1 is a necessary and sufficient mediator of inflammation and mediates inflammatory response by interacting with toll-like receptor (TLR)-2 and 4. HMGB-1 is involved in a series of diseases including sepsis, ischemia-reperfusion injury, neurological conditions, cardiovascular diseases, autoimmune diseases, endometriosis, and cancers (19). Recently, HMGB-1 has been implicated in endometriosis due to the important regulatory roles of inflammation in endometriosis (20). In 2016, for the first time, Bo and colleagues have demonstrated that inhibiting HMGB-1 suppresses the proliferation of HESCs, indicating that targeting HMGB-1 might be a strategy for endometriosis therapy (20). However, it is still unknown how inhibiting HMGB-1 could exert beneficial effects against endometriosis. Therefore, in the present study, we aimed to explore the roles of HMGB-1 in endometriosis and its underlying mechanisms.

## Materials and Methods

### Specimen Collection

This study was approved by the ethic committee of Quanzhou First Hospital Affiliated to Fujian Medical University. The participants have read and signed the informed consent. All surgeries were performed during the proliferative stage of the patients’ menstrual cycle, which was confirmed based on clinical or histologic criteria. This study enrolled participants at the age of 20 to 35, who had unilateral or bilateral ovarian chocolate cysts with a diameter ≥ 3 cm, as well as age-matched healthy volunteers. These participants were having regular menstrual cycles and had no history of hormonal treatment for at least 3 months prior to the current study.

In total, 58 participants with ovarian endometriosis were recruited in this study. Eutopic endometrium (n = 58) was collected when participants underwent laparoscopic treatment for infertility and/or ovarian cysts. Ectopic endometrium (n = 58) was carefully stripped from the inner cyst wall to avoid contamination with surrounding ovarian tissues. The ectopic endometrium was classified as stage III or IV, according to the revised American Fertility Society (AFS) classification. In addition, a group of healthy volunteers were recruited as the healthy control group and endometrial specimens (n= 20) were collected during hysteroscopy. Laproscopic examination was used to confirm that they did not have endometriosis. An endometrial specimen was collected from healthy fertile women who were undergoing laparoscopic tubal ligation or reversal of tubal sterilization by hysteroscopy.

In total, we collected ectopic endometrium (n = 58) and eutopic endometrium (n = 58) from the patients, and normal endometrium (n = 20) from the healthy volunteers. All specimens were immediately frozen and stored in liquid nitrogen for further assays.

### Immunohistochemistry Staining

We selected normal endometrial specimens (n = 8) from the healthy control group. Besides, we also selected ectopic endometrium (n = 8) and eutopic endometrium (n = 8) from patients with endometriosis for immunohistochemistry staining. Immunohistochemistry staining was performed according to previously reported method (21). After the tissues were collected, paraffin-embedded specimens from the endometriosis and control groups were sectioned at 7 μm, prepared with xylene and ethanol, pressurized, and heated with proteinase K for antigen retrieval. Next, endogenous peroxidase was eliminated and a primary antibody against HMGB-1 (1: 100, ProteinTech, Chicago, IL, USA) was added. After that, a secondary antibody was added and hematoxylin counterstaining was performed. The slides were observed and photographed under a microscope. ImageJ (version 5.0, Bio-Rad, USA) software was applied for semi-quantitative analysis. The immunohistochemical histological score (Hscore) was calculated by the formula [Hscore1/4 *P* (*Pi* x i)/100], according to previously reported method (22). *Pi* represents the percentage of positive cells for each intensity and staining intensity (*i*) represents the range of staining intensity. Pi was evaluated according to the following criteria: 0 indicates <5%, 1 indicates 5% to 25%, 2 indicates 25% to 50%, 3 indicates 50% to 75%, and 4 indicates >75%. *i* was evaluated according to a 4-point scale in the followings: 0 1/4 negative, 1 1/4 weak, 2 1/4 moderate, and 3 1/4 strong. There were two independent scorers in this study. The slides were blinded during scoring.

### HESC Isolation and HMGB-1 shRNA Transfection

HESCs were isolated from the ectopic endometrium of women with endometriosis (n = 5), according to a previously reported method (21). The isolated HESCs cells were cultured in Dulbecco’s modified Eagle’s/F12 medium containing 20% fetal bovine serum (FBS, Gibco) and 1% Penicillin-Streptomycin solutions (Gibco). As reported previously (21), the purity of isolated stromal cells was >95%, and stromal cells were contaminated by less than 1% of epithelial cells, as determined by diffuse and strong cytoplasmic immunostaining for vimentin and negative cellular staining for E-cadherin. When the cells reached 60-70% confluency, they were transfected with either HMGB-1 shRNA lentiviral vector or negative control vectors. HMGB1 shRNA lentiviral vector was constructed according to a previously reported method (23). The sequence of HMGB1 shRNA was 5’- GGA CAA GGC CCG TTA TGA A-3’. The sequence of control shRNA was 5’-TTC TCC GAA CGT GTA CGT -3’.

To determine the effects of HMGB1 knockdown on cell viability, cell survival was analyzed using a 3-(4,5-dimethylthiazol-2-yl)-2, 5-diphenyltetrazolium bromide (MTT) assay (Promega, Wisconsin, United States). HESCs were transfected with shNC or shHMGB1. Cell viability was measured at 0, 24h, 48h, and 72h after transfection. % cell viability = (mean absorbance in test wells)/(mean absorbance in control wells) × 100.

The transfected HESCs were seeded in petri dish, and fresh medium was applied to keep the cells healthy by providing nutrients before hypoxia treatment. The culture dishes were incubated in a modular incubator chamber containing humidified hypoxic air (1% O_2_, 5% CO_2_, and 94% N_2_) at 37°C. After the cells were cultured under the hypoxic conditions for 24 h, cells were collected for Western blotting and the supernatant was collected for ELISA.

### Quantitative Reverse Transcription PCR (RT-qPCR)

Normal endometrial specimens (n = 12) from the healthy control group, and ectopic endometrium (n = 35) and eutopic endometrium (n = 35) from patients with endometriosis were used for RT-qPCR. Total RNA was isolated from tissues or cells by using TRIzol reagent and the concentration of RNA was determined. The RNA quality was assessed by Nanodrop. The RNA was used to synthesize cDNA using a reverse transcription kit. After the reverse transcription reaction, the advanced master mix was used for quantitative analysis. To analyze the accuracy of the PCR reaction, the melt curves were used. To evaluate the expression of genes, the 2-^△△^Ct values were calculated using *GAPDH* as an internal control. *GAPDH* is known as the most common internal control in RT-qPCR and its expression can be detected in the tissues and cells used in this study. Therefore, we selected *GAPDH* as an internal control gene. The primers were listed below:

*HMGB-1* forward: 5’- GCT CAG AGA GGT GGA AGA CCA-3’, and reverse: 5’- GGT GCA TTG GGA TCC TTG AA-3’; *beclin-1* forward: 5’- CCA TGC AGG TGA GCT TCG T -3’, and reverse: 5’- GAA TCT GCG AGA GAC ACC ATC -3’; *GAPDH* forward: 5’- CCA TGC AGG TGA GCT TCG T -3’, and reverse: 5’- TGT CAT CAT ATT TGG CAG GTT T -3’.

### ELISA

Normal endometrial specimens (n = 12) from the healthy control group, and ectopic endometrium (n = 35) and eutopic endometrium (n = 35) from patients with endometriosis were used for ELISA. The levels of inflammatory cytokines (HMGB-1, IL-6, TNF-α, and IL-1β) in normal endometrium, eutopic endometrium and ectopic endometrium were determined using specific ELISA kits following the manufacturer’s instructions. The proteins were isolated from the endometrial specimens. In brief, collected endometrial tissues were washed three times with phosphate-buffered saline. Radioimmunoprecipitation assay buffer containing protease inhibitor was used to lyse the tissues or cells. The tissues were homogenized in the lysis buffer. After that, the lysis buffer was kept on ice for 30min, centrifuged at 12,000 g at 4°C for 15 min. The concentrations of proteins were quantified by using the bicinchoninic acid assay. ELISA kits of IL-6 (ab178013), TNF-α (ab181421), and IL-1β (ab215539) were purchased from Abcam (Cambridge, MA, USA) and HMGB1 (#MBS451177) was purchased from MyBioSource (San Diego, CA, USA). A standard curve was established in the ELISA assays. The concentrations of cytokines including IL-6, TNF-α, and IL-1β as well as HMGB1 were calculated based on the standard curve.

### Western Blotting

Normal endometrial specimens (n = 10) from the healthy control group, and ectopic endometrium (n = 10) and eutopic endometrium (n = 10) from patients with endometriosis were used for Western blotting. Collected endometrial tissues and cultured HESCs were washed three times with phosphate-buffered saline. Radioimmunoprecipitation assay buffer containing protease inhibitor was used to lyse the tissues or cells. The tissues were homogenized in the lysis buffer. The cells were scraped in the lysis buffer. After that, the lysis buffer was kept on ice for 30 min, centrifuged at 12,000 g at 4°C for 15 min and diluted in 5× sample loading buffer (Beyotime Biotechnology, Shanghai, China). The bicinchoninic acid assay was used to quantify the concentrations of proteins. An equal amount of protein samples was loaded into each well of the SDS-PAGE gel followed by transferring the proteins onto the PVDF membrane. After the membrane was blocked in 5% bovine serum albumin solution, the primary antibodies against HMGB-1 [1:1000, Cell Signaling Technology (CST)], Beclin1 (1:1000, CST), LC3 (1:1000, Abcam), Atg13 (1:1000, Abcam), p62 (1:1000, Abcam) and GAPDH (1:1000; Abcam) were respectively added and incubated overnight at 4°C. Next, appropriate secondary antibodies were applied and incubated for 2 hours at room temperature. An ECL detection kit was applied for chemiluminescence development. The imaging system was applied to quantify the expression of the target proteins.

### Statistical Analysis

Statistical analysis was performed with GraphPad Prism8 (San Diego, CA, United States). Data were presented as mean ± SD. Spearman’s rank correlation analysis was employed to analyze the correlations between target genes. One-way ANOVA followed by Dunnett’s T3 multiple comparison test or Mann Whitney test was performed for data analysis. A *p*-value less than 0.05 was defined as statistical significance between two groups.

## Results

### The Expression Patterns of HMGB-1 in the Endometrial Tissues

**Figure 1:**
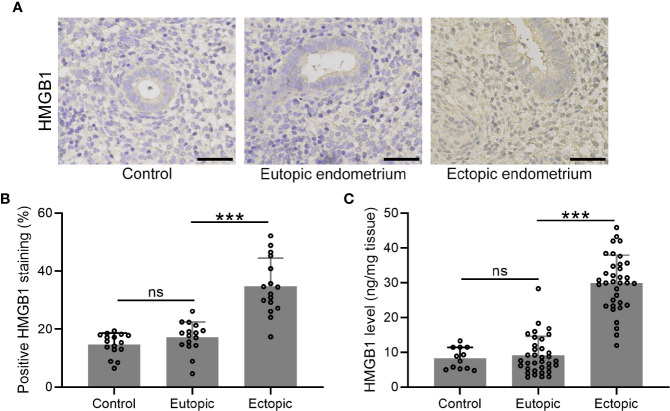
The expression patterns of HMGB1 in the endometrial tissues. **(A)** Immunohistochemical staining showed the protein localization of HMGB1 in normal endometrium, eutopic endometrium, and ectopic endometrium (magnifications of 200×, scale bar: 50 μm). **(B)** The positive HMGB staining in each group was qualified using ImageJ (n = 15). **(C)** The protein levels of HMGB1 in these groups were determined using specific ELISA (n=12 for the control group and n= 35 for eutopic and ectopic groups). The data were expressed as means ± SD. One-way ANOVA followed Dunnett’s T3 multiple comparisons test was performed. ***P < 0.001 indicates significant difference. ns indicates no significant difference.

We determined the expression and localization of HMGB-1 in the endometrial tissues including normal, eutopic, and ectopic endometria. The results showed elevated HMGB-1 in the ectopic endometrium (**Figures 1A, B**). Interestingly, we did not observe that eutopic endometria of women with and without endometriosis did not show any differences (**Figure 1B**). Consistently, the level of HMGB-1 in the ectopic endometrium was significantly increased as compared to the normal and eutopic endometrial tissues (**Figure 1C**).

### The Levels of Inflammatory Cytokines and Their Relationships With HMGB-1 in the Endometrial Tissues

**Figure 2:**
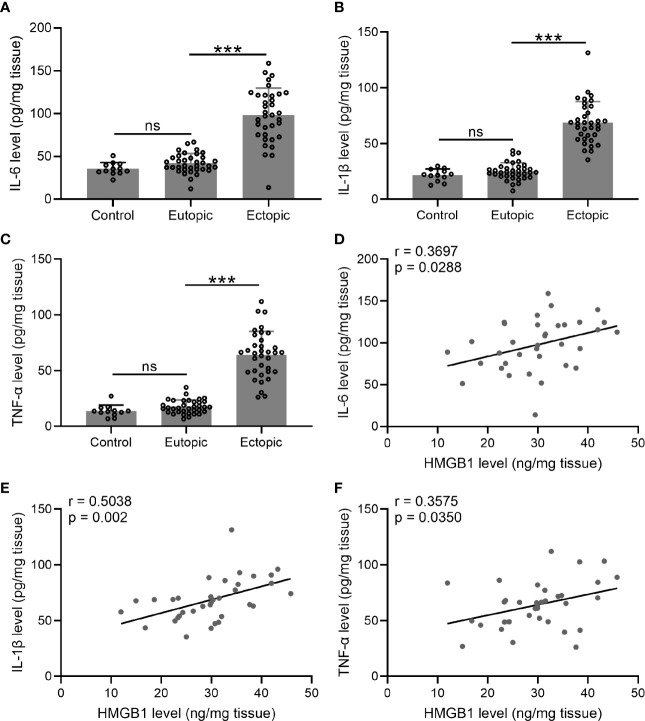
The levels of inflammatory cytokines (IL-6, TNF-α, and IL-1β) and their relationships with HMGB1 in the endometrial tissues. **(A–C)** The levels of inflammatory cytokines including IL-6, IL-1β, and TNF-α were determined using specific ELISAs in different groups (n = 12 for the control group and n = 35 for eutopic and ectopic groups). **(D–F)** The correlations of HMGB1 and inflammatory cytokines (IL-6, IL-1β, TNF-α) were determined using Spearman’s rank correlation analysis (n=35). The data were expressed as means ± SD. One-way ANOVA followed Dunnett’s T3 multiple comparisons test was performed. ***P < 0.001 indicates significant difference. ns indicates no significant difference.

We next investigated the levels of inflammatory cytokines (IL-6, TNF-α, and IL-1β) in the endometrial tissues. Interestingly, we found that the levels of IL-6, TNF-α, and IL-1β were significantly increased in the ectopic endometrium as compared to the normal and eutopic endometrial tissues (**Figures 2A–C**). The relationship between inflammatory cytokines and HMGB-1 was explored using Spearman’s rank correlation analysis. The results showed strong positive correlations between HMGB-1 and all three inflammatory cytokines (**Figures 2D–F**).

### The Levels of HMGB-1 and Beclin 1 and Their Relationships in the Endometrial Tissues

**Figure 3:**
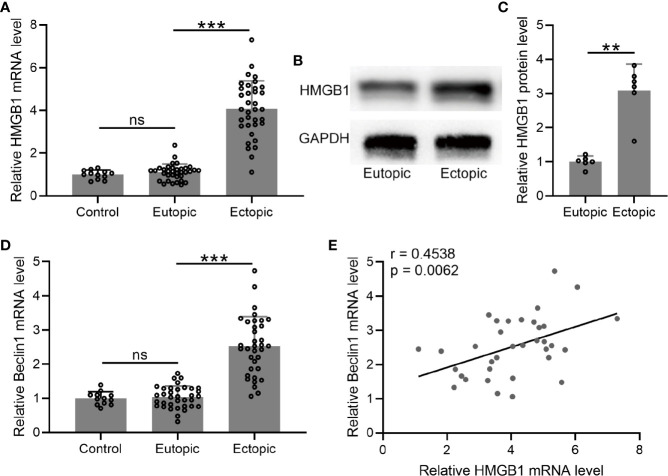
The levels of HMGB1 and Beclin 1 and their relationships in the endometrial tissues. **(A–D)** The mRNA and protein levels of HMGB1 were determined using qRT-PCR and western blotting, separately, in normal endometrium, eutopic endometrium, and ectopic endometrium. The mRNA levels of Beclin 1 were determined using qRT-PCR in normal endometrium, eutopic endometrium, and ectopic endometrium (n = 12 for the control group and n = 35 for eutopic and ectopic groups). The experiments were repeated independently for three times. **(E)** The correlations of HMGB1 and Beclin 1 were determined using Spearman’s rank correlation analysis (n=35). The data were expressed as means ± SD. T test followed Mann Whitney test was performed in **(A, D)**. One-way ANOVA followed Dunnett’s T3 multiple comparisons test was performed in **(C)**. **P < 0.01, ***P < 0.001 indicate significant difference. ns means no significance.

We determined the mRNA and protein levels of HMGB-1 in the endometrial tissues, which were significantly increased in the ectopic endometrium as compared to the normal and eutopic endometrial tissues (**Figures 3A–C**).

Next, we measured the mRNA level of beclin-1 in the endometrial tissues. The results demonstrated that the mRNA level of beclin-1 was significantly increased in the ectopic endometrium as compared to the normal and eutopic endometrial tissues (**Figure 3D**). In addition, we explored the relationship between beclin-1 and HMGB-1, and identified a positive correlation between these two factors in the ectopic endometrium (**Figure 3E**).

### Autophagy-Related Proteins Were Upregulated in Ectopic Endometrium

**Figure 4:**
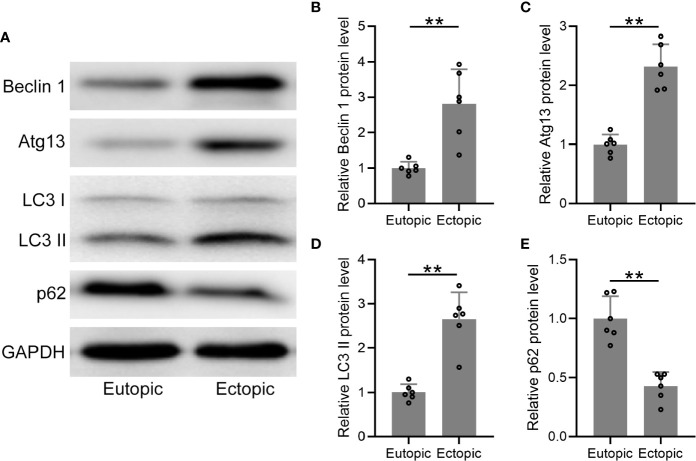
The protein levels of autophagy-related proteins were elevated in the ectopic endometrium. **(A)** Representative illustrations of Western blotting showed the protein expressions of Beclin 1, Atg 13, LC3-I, LC3-II, and p62 in eutopic endometrium and ectopic endometrium. **(B–E)** The expression levels of Beclin 1, Atg 13, LC3-I, LC3-II, and p62 in eutopic endometrium and ectopic endometrium were qualified using ImageJ (n = 4). The expressions were normalized to GADPH. The data were expressed as means ± SD. Student t test followed Mann Whitney test was performed. **P < 0.01 indicates significant difference.

We further determined the protein levels of autophagy-related markers (beclin-1, atg13, LC3, and p62) in the eutopic and ectopic endometrial tissues (**Figure 4A**), which were found to be significantly increased in the ectopic endometrial tissues (**Figures 4B, C**). The conversion of LC3-I to the lower migrating form LC3-II is considered as an indicator of autophagy. In the present study, more membrane-bound LC3-II was observed in the ectopic endometrium than in the eutopic endometrium (**Figure 4D**). In addition, the protein level of p62 was significantly decreased in the ectopic endometrial tissues (**Figure 4E**). Taken together, autophagy-related proteins were upregulated in the ectopic endometrium.

### Transfection of shHMGB-1 Reduced HMGB-1 Expression in HESCs From Endometriosis Patients

**Figure 5:**
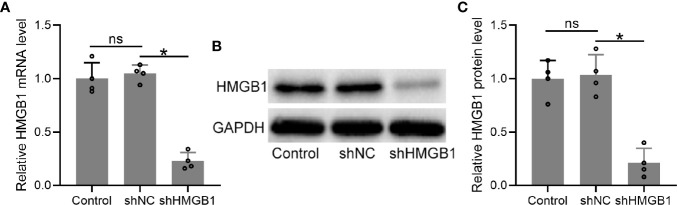
Transfection of shHMGB1 knocked down the levels of HMGB1 in human endometrial stromal cells (HESCs) from endometriosis patients. **(A–C)** The HESCs were isolated from the ectopic endometrium. Next, the cells were transfected with shNC or shHMGB1 for 24 h. The mRNA and protein levels of HMGB1 were determined using qRT-PCR (n = 4) and Western blotting, separately, in the HESCs. The experiments were repeated independently for three times. The expressions were normalized to GADPH. The data were expressed as means ± SD. One-way ANOVA followed Dunnett’s T3 multiple comparisons test was performed. *P < 0.05 indicates significant difference. ns indicates no significant difference.

To explore the roles of HMGB-1 in endometriosis, we knocked down HMGB-1 in the HESCs by using a lentiviral vector carrying HMGB-1 shRNA. The results demonstrated that HMGB-1 knockdown did not affect cell viabilities of HESCs (**Figure S1**). The mRNA levels of HMGB-1 in the HESCs were significantly decreased following transfection with shHMGB-1 (**Figure 5A**). Similarly, the protein levels of HMGB-1 were also significantly decreased (**Figures 5B, C**). Taken together, these results demonstrated that HMGB-1 was successfully silenced in the HESCs.

### HMGB-1 Silencing Suppressed Inflammatory Cytokines in the HESCs Under Hypoxic Condition

**Figure 6:**
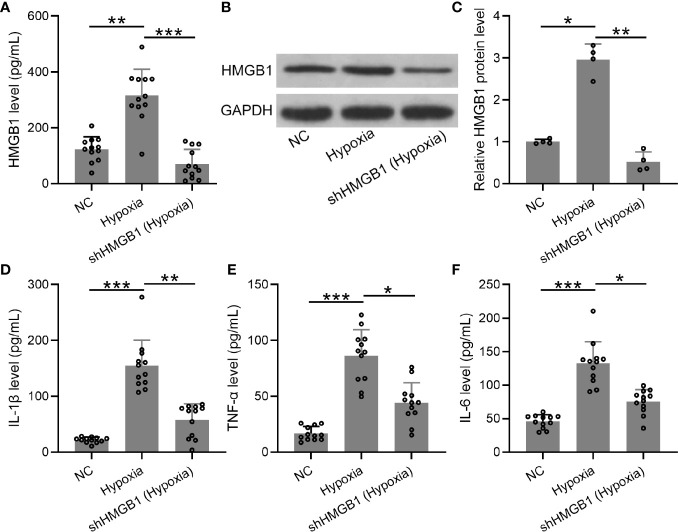
The knockdown of HMGB1 suppressed inflammatory cytokines in the HESCs under hypoxic conditions. **(A–C)** The HESCs were transfected with shNC or shHMGB1 for 24 h followed by incubating under hypoxic conditions for another 24 h. The secreted and cellular HMGB1 were determined using ELISA and Western blotting, separately (n = 12). The expressions were normalized to GAPDH. **(D–F)** The levels of inflammatory cytokines (IL-6, TNF-α, and IL-1β) were determined using specific ELISAs (n = 12). The HESCs that were transfected with shNC were cultured in normoxia. The data were expressed as means ± SD. One-way ANOVA followed Dunnett’s T3 multiple comparisons test was performed. *P < 0.05, **P < 0.01, and ***P < 0.001 indicate significant difference.

Furthermore, we explored the levels of inflammatory cytokines in HESCs transfected with shHMGB-1 under hypoxic conditions. First, mRNA and protein levels of HMGB-1 were significantly decreased in HESCs transfected with shHMGB-1 (**Figures 6A–C**), indicating that HMGB-1 was successfully silenced in the transfected HESCs under hypoxic conditions. Next, significantly increased inflammatory cytokines (IL-6, TNF-α, and IL-1β) were observed in HESCs under hypoxic conditions, whereas shHMGB-1 transfection significantly suppressed these inflammatory cytokines in the HESCs. These results suggested that HMGB-1 silencing suppressed inflammatory cytokines in HESCs under hypoxic conditions (**Figures 6D–F**). It should be noted that shNC did not affect the production of HMGB-1, IL-6, TNF-α, and IL-1β in hypoxic condition (**Figure S2**).

### HMGB1 Silencing Regulated Autophagy in HESCs Under Hypoxic Condition

**Figure 7:**
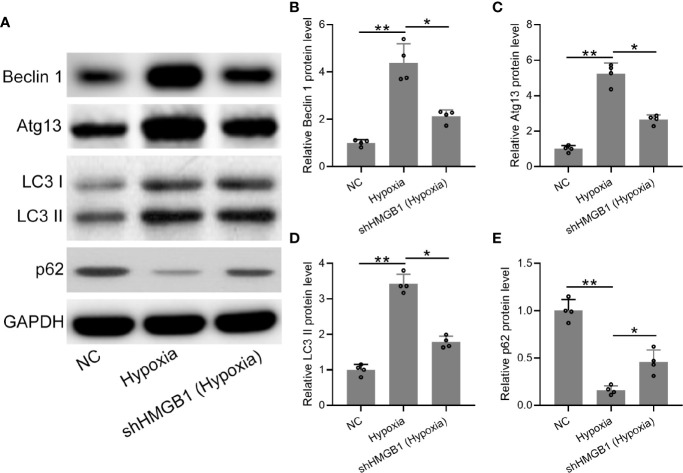
The knockdown of HMGB1 regulated autophagy in the HESCs under hypoxic conditions. **(A)** The HESCs were transfected with shNC or shHMGB1 for 24 h followed by incubating under hypoxic conditions for another 24 h. The Representative illustrations of Western blotting showed the protein expressions of Beclin 1, Atg 13, LC3-I, LC3-II, and p62. **(B–E)** The expression levels of Beclin 1, Atg 13, LC3-I, LC3-II, and p62 in the shHMGB1-transfected HESCs under hypoxia conditions (n = 4). The HESCs that were transfected with shNC were cultured in normoxia. The expressions were normalized to GAPDH. The data were expressed as means ± SD. One-way ANOVA followed Dunnett’s T3 multiple comparisons test was performed. *P < 0.05 and **P < 0.01 indicate significant difference.

Finally, we explored the regulatory roles of HMGB-1 in autophagy under hypoxia conditions. The protein expression levels of autophagy-related markers (LC3, beclin-1, atg13, and p62) were determined (**Figure 7A**). We found that protein levels of beclin-1 and atg13 were significantly decreased in the shHMGB-1 transfected HESCs under hypoxic conditions (**Figures 7B, C**). Besides, less membrane-bound LC3-II was observed in the shHMGB-1 transfected HESCs (**Figure 7D**). In addition, under hypoxic condition, the protein level of p62 was significantly increased in the shHMGB-1 transfected HESCs as compared to control HESCs (**Figure 7E**). These results suggested that HMGB-1 silencing regulated autophagy in HESCs under hypoxic conditions.

## Discussion

In the present study, we investigated the roles of HMGB-1 in endometriosis and its underlying mechanisms. Our results revealed that HMGB-1 was upregulated in ectopic endometrium, which was also positively correlated with inflammatory cytokines IL-6, TNF-α, and IL-1β, as well as autophagy-related protein beclin-1, suggesting that HMGB-1 might be involved in endometriosis. To confirm this hypothesis, HMGB-1 was silenced in HESCs, which consequently suppressed inflammatory cytokines and regulated autophagy under hypoxia. These results suggest that HMGB-1 contributes to endometriosis in part by regulating inflammatory response and autophagy, therefore targeting HMGB-1 might be an effective strategy for endometriosis therapy.

Endometriosis is a common chronic inflammatory condition (24). Inflammation plays an important role in the pathogenesis of endometriosis. The inflammatory cytokines and mediators accelerate the abnormal growth and infiltration of endometrial cells, including endometrial epithelial cells and stromal cells, into the deep endometriosis tissues including ovaries and the pelvic peritoneum, followed in order of decreasing frequency by deep lesions of the pelvic subperitoneal space, the intestinal system, and the urinary system (24, 25). Non-steroidal anti-inflammatory drugs are the common first-line treatment for endometriosis (26). HMGB-1 is a key mediator that plays an important role in the activation of inflammatory responses (18, 19, 27) and also serves as ligands of TLR2 and TLR4, which are responsible for recognizing pathogen-associated molecular patterns (19). HMGB-1 is commonly used as an admissible biomarker for endometriosis (28). Bo and colleagues demonstrated that inhibiting HMGB-1 suppressed the proliferation of HESCs in 2016 (20), indicating that targeting HMGB-1 might be a strategy for endometriosis therapy. In the present study, we firstly investigated the expression patterns of HMGB-1 in normal, eutopic, and ectopic endometrial tissues, and found that HMGB-1 was significantly upregulated in the ectopic endometrium as compared to the normal and eutopic endometrium.

HMGB-1 has been extensively studied to understand the pathogenesis of inflammatory and autoimmune diseases (29) including endometriosis (20). It binds to TLR4 and induces sterile inflammation by a cascade involving the nuclear factor (NF)-κB pathway in the human endometrium (20). To confirm that the role of HMGB-1 in endometriosis was associated with inflammatory cytokines, we analyzed the correlations between HMGB-1 and inflammatory cytokines in HESCs. Previous studies have demonstrated that the levels of pro-inflammatory cytokines IL-6, TNF-α, IL-1β, and IL-17 in the localized lesion (ectopic endometrium and peritoneal cavity) and circulation (serum) were significantly increased in women with endometriosis (30, 31). As expected, in the present study, we observed that inflammatory cytokines including IL-6, TNF-α, and IL-1β were indeed significantly increased in the ectopic endometrium. In addition, we also observed strong positive correlations between HMGB-1 and these inflammatory cytokines. As discussed, HMGB-1 is known as a mediator for triggering inflammation (29). Therefore, we speculated that elevated HMGB-1 could trigger inflammatory responses, leading to increased production of inflammatory cytokines.

Autophagy has been reported to play an important role in endometriosis (13), which is activated when cells undergo stresses such as endoplasmic reticulum stress and nutrient deprivation (13). A previous study demonstrated that autophagy was suppressed in eutopic and ectopic endometria from endometriosis patients, and also revealed a relationship between autophagy and endometriosis (32). Another study reported that autophagy was suppressed in HESCs. Importantly, autophagy is reported to affect endometriosis in part *via* regulating the proliferation and apoptosis of HESCs (16). Consistently, our results demonstrated the abnormal expression of autophagy-related markers beclin-1, atg13, and p62 in the ectopic endometrium. In addition, the conversion of LC3-I to the lower migrating form LC3-II is used as an indicator of autophagy. In this study, our results revealed abundant membrane-bound LC3-II in the ectopic endometrium.

Endogenous HMGB-1 is known as a critical regulator of autophagy, as HMGB-1 translocation induces autophagy after prolonged cellular stress (33, 34). Moreover, targeted ablation of HMGB-1 increases apoptosis and inhibits autophagy by sustaining the interaction between beclin-1 and B-cell lymphoma 2. Interestingly, a previous study reported that HMGB1 regulated autophagy during chemotherapy in endometrial carcinoma cells (35). However, the potential relationship between HMGB-1 and autophagy in endometriosis remains uninvestigated. In this study, we found a positive correlation between HMGB-1 and beclin-1 in the ectopic endometrium. Interestingly, we noticed that HMGB-1 silencing also regulated the expression of autophagy-related proteins beclin-1, LC3, atg13, and p62.

Hypoxia is a physiological condition occurring in the endometrial tissues and is also known as a key contributing factor of endometriosis (36). Upregulated autophagy is observed in the ectopic endometrium of patients with ovarian endometriosis (37). However, it is still unclear whether autophagy could be activated in HESCs under a hypoxic environment. Therefore, to confirm the regulatory role of HMGB-1 in autophagy during endometriosis, hypoxia treatment was used to induce autophagy in HESCs. Meanwhile, to confirm the regulatory role of HMGB-1 in endometriosis, we silenced HMGB-1 in HESCs under hypoxic conditions. Surprisingly, HMGB-1 silencing suppressed inflammatory cytokines IL-6, TNF-α, and IL-1β and regulated autophagy-related proteins beclin-1, LC3, atg13, and p62 in the HESCs under hypoxic condition. These results suggest that targeting HMGB-1 might be an effective strategy for the treatment of endometriosis through regulating inflammation and autophagy. A previous study reported that hypoxia treatment upregulated HMGB-1 in macrophages by activating extracellular signal-regulated kinase and c-Jun N-terminal kinase (38). In the present study, we also observed that hypoxia treatment upregulated the expression of HMGB-1 in HESCs, consistent with previous study. However, the underlying mechanism by which hypoxia regulates HMGB-1 should be further investigated in future studies. In addition, autophagy is known to be upregulated in endometriosis and hypoxia is a useful way to induce autophagy (16). Therefore, the present study was designed to investigate the inhibition of HMGB1 on autophagy in hypoxic conditions. However, the effects of inhibition of HMGB1 on autophagy in normoxia conditions should be investigated in further studies.

## Conclusion

The present study revealed elevated HMGB-1 in the ectopic endometrium, as well as positive correlations between HMGB-1 and levels of inflammatory cytokines and autophagy-related protein in the endometrial tissues. HMGB-1 silencing significantly downregulated the inflammatory cytokines and regulated autophagy-related markers under hypoxic conditions. These results demonstrated that HMGB-1 might contribute to endometriosis in part by regulating inflammatory response and autophagy.

## Data Availability Statement

The raw data supporting the conclusions of this article will be made available by the authors, without undue reservation.

## Ethics Statement

The studies involving human participants were reviewed and approved by Quanzhou First Hospital Affiliated to Fujian Medical University. The patients/participants provided their written informed consent to participate in this study.

## Author Contributions

Data collection and analysis: JH, XC, and YL. Study designed and manuscript writing: YL. All authors contributed to the article and approved the submitted version.

## Funding 

The study was supported by the Quanzhou science and technology plan project (2018Z073).

## Conflict of Interest

The authors declare that the research was conducted in the absence of any commercial or financial relationships that could be construed as a potential conflict of interest.
