# Supplementary material for: HMGB1 Mediated Inflammation and Autophagy Contribute to Endometriosis
Source: Front Endocrinol (Lausanne). 2021 Mar 19;12:616696. doi: 10.3389/fendo.2021.616696 (PMC8018282; doi:10.3389/fendo.2021.616696)
Supplement: Supplementary file 1 [file DataSheet_1.pdf]

Supplementary Materials

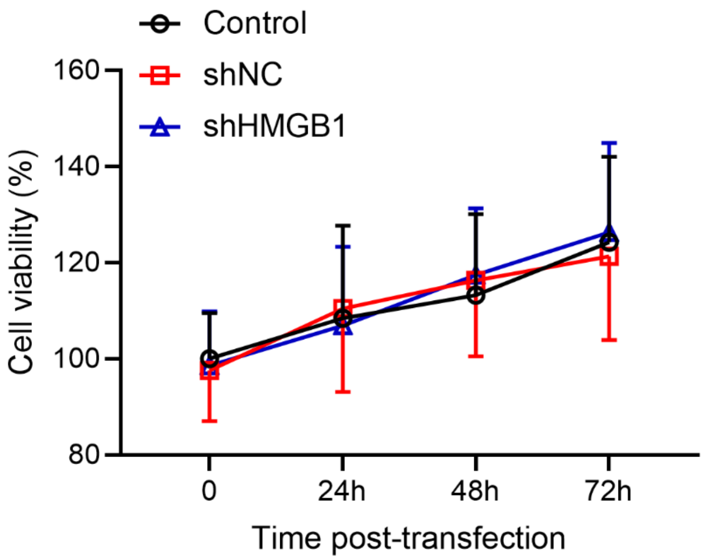

Figure S1. HMGB1 knockdown in HESCs did not affect cell viability. HESCs were transfected with shNC or shHMGB1, and cell viability was measured at 0, 24 h, 48 h, and 72 h after transfection (n =6). Data were presented as means ± SD.

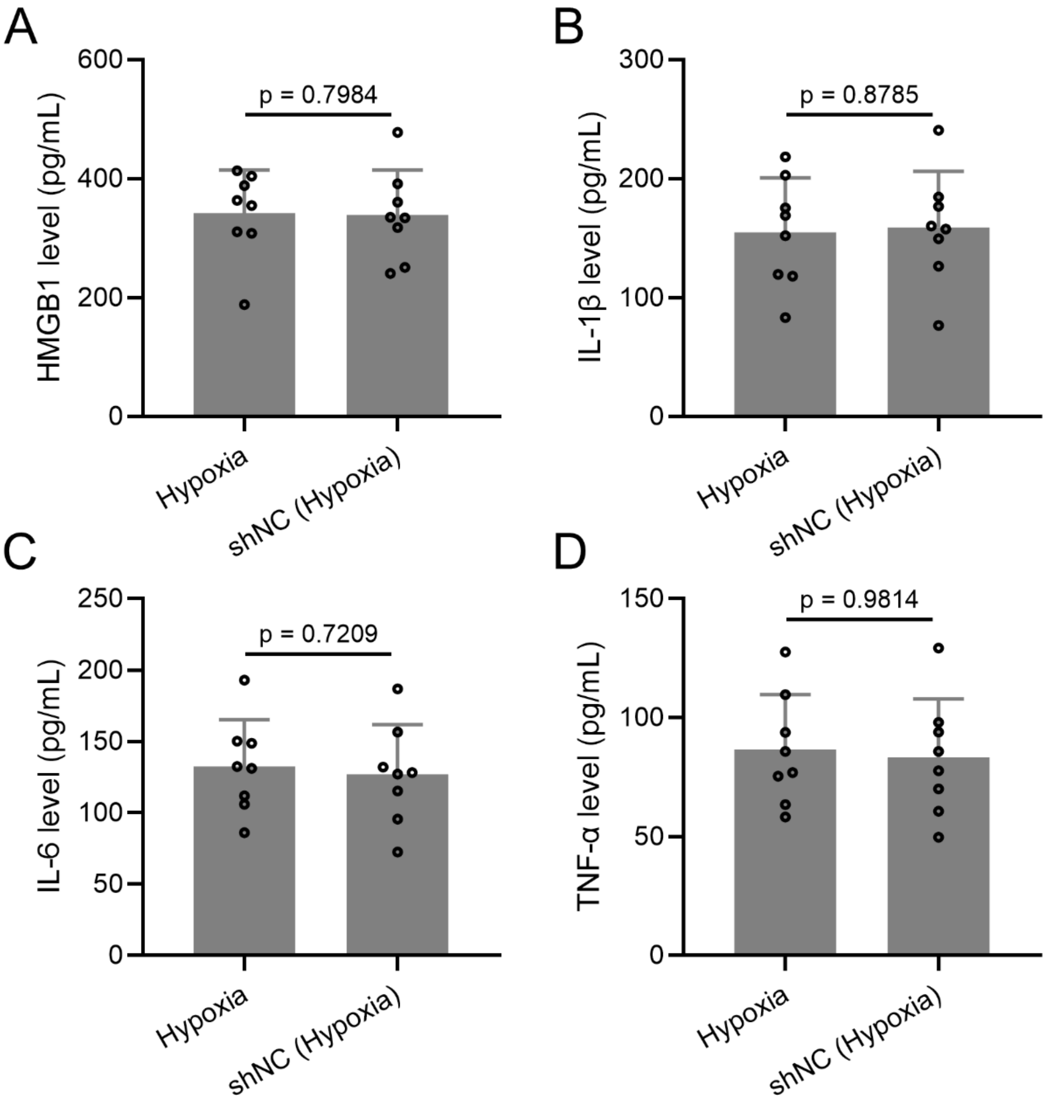

Figure S2. HESCs were transfected with shNC or not for 24 h and then incubated in hypoxic conditions for another 24 h. ELISA was used to measure the HMGB1 (A), IL-1β (B), IL-6 (C) and TNF-α (D) concentrations in the cell supernatants. n = 8 for each group. Mann Whitney test.
